# Evidence Against Novelty-Gated Encoding in Serial Recall

**DOI:** 10.5334/joc.207

**Published:** 2022-02-08

**Authors:** Klaus Oberauer, Simon Farrell, Christopher Jarrold, Marcel Niklaus

**Affiliations:** 1 Department of Psychology, University of Zurich, Switzerland; 2 School of Psychological Science, University of Western Australia, Australia; 3 School of Psychological Science, University of Bristol, United Kingdom

**Keywords:** Working memory, Short-term memory, Mathematical modelling

## Abstract

Novelty-gated encoding is the assumption that events are encoded more strongly into memory when they are more novel in comparison to previously encoded events. It is a core assumption of the SOB model of serial recall (Farrell & Lewandowsky, 2002). We present three experiments testing some predictions from novelty-gated encoding. Experiment 1 shows that the probability of recalling the third item in a list correctly does not depend on whether it is preceded by phonologically similar or dissimilar items. Experiment 2 shows that in lists of items from three classes (nonwords, spatial locations, and abstract drawings) the probability of recalling an item does not depend on whether it is preceded by items from the same or another class. Experiment 3 used a complex-span paradigm varying the phonological similarity of words that are read aloud as distractors in between memory items. Contrary to a prediction from novelty-gated encoding, similar distractors did not impair memory more than dissimilar distractors. The results question the assumption of novelty-gated encoding in serial recall. We discuss alternative explanations for the phenomena that this assumption has previously helped to explain. The present evidence against novelty-gated encoding might point to boundary conditions for the role of prediction error in the acquisition of memories.

The serial-recall task is one of the main experimental work horses for studying short-term or working memory. In this task, participants are given a list of items and are asked to recall them – usually immediately after presentation – in their given order. Behavior in this task has been studied in great detail (for a review see Hurlstone, Hitch, & Baddeley, 2014), and it has been the target of explanation for several computational models (for a review see Lewandowsky & Farrell, 2008b). A variant of serial recall, the complex-span task, is the most often used task for measuring individual differences in working-memory capacity (Conway et al., 2005; Daneman & Carpenter, 1980). In complex-span tasks, presentation of the list item alternates with brief periods of a concurrent task that involves processing of distractor stimuli (e.g., solving arithmetic problems, or reading sentences).

Through an analysis of error patterns and recall times, Farrell and Lewandowsky (2004) identified a *primacy gradient* as one of the features that a successful model of serial recall probably needs. A primacy gradient means that memory strength declines from the first list item to the last. Such a primacy gradient facilitates recall of a list in forward order, particularly when combined with response suppression – the suppression of items after they have been recalled. This is because at the beginning of recall the first item is the most strongly activated and therefore has a good chance of winning the competition for output. After this first item has been recalled and suppressed, the second list item is the strongest, so that it is likely to win the competition next, and so on (Grossberg & Stone, 1986; Page & Norris, 1998). Here we are concerned with one mechanism for how such a primacy gradient could come about: Novelty-gated encoding.

Novelty-gated encoding (a.k.a. energy-gated encoding) means that events are encoded into memory more strongly when they are more novel, where novelty is assessed with respect to the current contents of memory. The rationale for this idea is that an efficient memory system should preferentially encode information that cannot yet be predicted by already acquired knowledge. As such, the novelty-gated encoding hypothesis has much in common with the assumption that associative learning (Wills, 2009), but also the acquisition of declarative, episodic memory, is driven by prediction error (Greve, Cooper, Kaula, Anderson, & Henson, 2017; Rouhani, Norman, & Niv, 2018). Novelty-gated encoding has been proposed as a mechanism in the SOB (serial-order in a box) model of serial recall (Farrell & Lewandowsky, 2002), and has been a core assumption of that model through several updates (Farrell, 2006; Lewandowsky & Farrell, 2008b; Oberauer, Lewandowsky, Farrell, Jarrold, & Greaves, 2012). It gives rise to a primacy gradient if we assume that at the beginning of encoding a list, there is no memory content related in any way to the list items, and that successive items on a list are typically at least somewhat similar to each other. Under these conditions, the first item is maximally novel and is encoded with maximal strength. The second item is less novel because of its similarity to the first item. Every successive item is less and less novel because it shares some features with all the previously encoded items, so that its combined similarity with all previously encoded items increases with the number of preceding list items. The more similar the list items are to each other, the more steeply memory strength declines over list position. This explains the negative effect of similarity on recall performance that has been observed for phonologically (Conrad & Hull, 1964) and visually similar lists (Logie, Della Sala, Wynn, & Baddeley, 2000).

Farrell and Lewandowsky (2003) present evidence for a new prediction that SOB makes by virtue of its novelty-gated encoding assumption: When phonologically similar and dissimilar items are mixed in a memory list, the dissimilar items on the mixed list are recalled better than the same items in a list that consists only of dissimilar items (see Farrell, 2006; Lewandowsky & Farrell, 2008a, for replications and extensions of this finding). In SOB, this effect arises because novelty gating means that the similar items in the mixed list are encoded more weakly than are dissimilar items that occur in the same list positions of the purely dissimilar list. Therefore, the dissimilar items in the mixed list have, on average, weaker competitors for recall, so that they are less likely to be confused with other list items. This mixed-similarity benefit is a unique prediction of SOB.

In an extension of the SOB model to the complex-span paradigm (Oberauer & Lewandowsky, 2008; Oberauer et al., 2012) novelty-gated encoding has been used to explain how much processing of the distractors in a complex span task interferes with recall of the memory items: When the distractors presented within a processing episode between two memory items are all similar to each other – in the extreme case, all identical – then all but the first distractor stimulus is encoded only weakly into memory, compared to processing episodes with different distractors. Because distractors encoded into memory create interference, encoding them more weakly results in less interference, and better memory. Lewandowsky, Geiger, and Oberauer (2008) and Lewandowsky, Geiger, Morrell, and Oberauer (2010) tested and confirmed this prediction: Participants had to remember lists of letters, and in between read words aloud as the distractor task. When these processing episodes involved reading four different words, memory was worse than when they had to read the same word aloud four times.

Here we present three experiments that tested three new predictions derived from the assumption of novelty-gated encoding. In Experiment 1 we test the prediction that an item in the middle of a list is recalled worse when it is preceded by, rather than followed by items that are phonologically similar to it. In Experiment 2 we investigate similarity on a coarser scale, testing the prediction that items are recalled worse when preceded by another item from the same broad category of stimuli than when preceded by items from other categories. With Experiment 3, we test whether distractors in a complex-span task are less damaging when they are phonologically similar to each other than when they are dissimilar. In all three experiments we found evidence against these predictions. The raw data of the three experiments are available on the OSF: *osf.io/7cjxv/*.

The three experiments were not planned jointly – rather, they were carried out separately at different times, and in part in different labs, and therefore differ in many details. What they have in common is that they were designed as tests of the novelty-gated encoding assumption as built into SOB. Together they convinced us that the assumption of novelty-gated encoding is probably in need of revision. We therefore decided to publish the accumulated evidence from these three experiments, as we do not wish to withhold evidence against a core assumption in one of our models.

## Experiment 1

**Figure 1:**
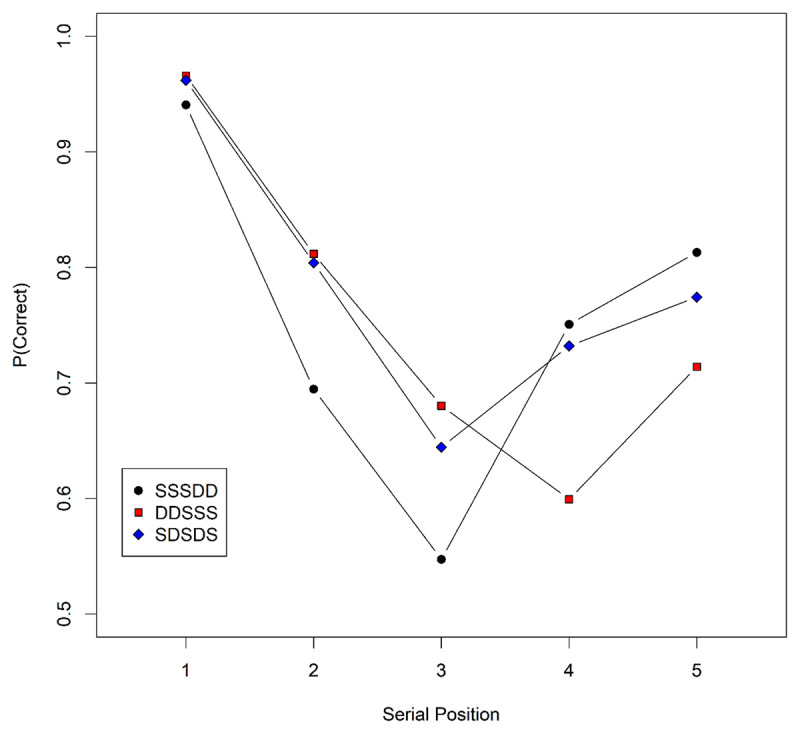
Serial-position curves for the three conditions of Experiment 1 as predicted by SOB-CS; the condition legend denotes the list composition of dissimilar (D) and similar (S) words. The predictions were generated by simulating 30 participants, with 100 trials per condition and participant, using the standard parameter values (Oberauer et al., 2012), except for the similarities between similar (S) words (set to 0.7 instead of the standard value of 0.65). This change was made so that the predicted main effect of similarity in positions 1, 2, 4, and 5 is approximately as large as in the data of Experiment 1.

In Experiment 1 we compared serial recall of three types of mixed-similarity lists. The lists consisted of five nouns that were either phonologically similar (S) to other nouns on the list, or dissimilar (D) to all other list items. The three list types all contain three S and two D items, and differed in how these were ordered: (1) DDSSS, (2) SSSDD, and (3) SDSDS. Novelty-gated encoding leads to the prediction that the third list item – which was always a similar item – is recalled worst in condition (2) and best in condition (1). This is because with list structure SSSDD, the third item is preceded by two items similar to it, so its encoding is substantially dampened through novelty gating. With list structure DDSSS, the third item is dissimilar to the preceding two items (it is only similar to the two items following it), and is therefore encoded relatively strongly. In the alternating structure SDSDS, the third list item’s encoding strength is somewhat dampened through its similarity to the first item, so that it should be recalled with a success intermediate between the other two conditions. We verified these predictions through simulations with SOB-CS (Oberauer et al., 2012); the simulated data are plotted in ***Figure 1***.

### Method

#### Participants

Thirty-one participants were recruited from the participant pool of the University of Bern, or through personal contact (16 female and 15 male). For this and the subsequent experiments, we chose the sample size on the basis of experience with similar studies that have provided unambiguous evidence for effects of about the size that we predicted from SOB-CS for the present experiments. The mean age of participants was 22 years (SD = 3.9). After being informed about the procedure, participants filled in a consent form. The study was approved by the ethics committee of the Faculty of Human Sciences of the University of Bern.

#### Materials

The list items were drawn from ten sets of six phonologically similar bisyllabic German nouns (***Table 1***). To create these sets, the phonological distances^1^ between 2500 nouns retrieved from Webcelex (*http://celex.mpi.nl*) were calculated. A non-hierarchical cluster analysis (partitioning around medoids as implemented in the pam package for R) was used to group the words into similar sets; the resulting sets – manually corrected for words that did not sound similar when spoken in the Swiss German dialect – had an average distance of .27, compared to an average between-set distance of .45.

**Table 1:** Words Used in Experiment 1.


Wetter	Rahmen	Filter	Anlass	Karte	Kosten	Theke	Weiche	Kumpel	Verband

Fenster	Graben	Silber	Ballast	Kante	Morgen	Ernte	Weizen	Rummel	Festland

Rechner	Rasen	Gitter	Anlauf	Farbe	Kolben	Rennen	Zeichen	Kummer	Tonband

Treffer	Braten	Finger	Applaus	Marke	Knochen	Sehne	Saite	Pumpe	Bestand

Becher	Rate	Ziffer	Inland	Taste	Quote	Rente	Leine	Muskel	Fahrbahn

Messer	Sahne	Winter	Atlas	Partner	Knoten	Steppe	Reihe	Tunnel	Verdacht


*Note*: Each column contains six phonologically similar words; words in different columns are phonologically dissimilar.

Lists for each trial were composed by first selecting three of the ten word sets at random (designated sets 1, 2, and 3). We then sampled three S words from set 1, and one D word each from sets 2 and 3. The S and D words were assigned at random to the S and D slots, respectively, of the desired list type.

#### Procedure

Each trial began with the presentation of a fixation cross for 0.65 s, followed by a 0.1 s blank screen. Five words were then shown one after another for 0.65 s followed by a blank screen that lasted 100 ms. The presentation rate of words was chosen to be high in order to provoke an adequate number of errors. Words were presented in 100 point Arial font, centered on the screen. In SOB, recall is modelled as selection from a set of candidates. Here we used a test procedure in which the experimenter defines the candidate set, thereby improving experimental control compared to conventional recall tests in which each person defines their own candidate set. Following presentation of the list words, a recall screen including a grid of boxes containing the candidate set of 15 words was presented. The candidate set consisted of five words each from the word sets 1, 2, and 3, including the list words. Participants were told to select the to-be-remembered words in the order they had been presented before. This procedure allowed no omissions or intrusions of non-candidate set words. There was no time limit and no corrections were possible.

The experiment was self-paced as the next trial was started by clicking on a go-box that appeared after five words had been selected. There were 30 trials per condition, presented in random order with the restriction of no more than three consecutive repetitions of the same condition. Two practice trials with phonologically different words from a different word-list preceded the main experiment. The experiment lasted about one hour.

### Results and Discussion

**Figure 2:**
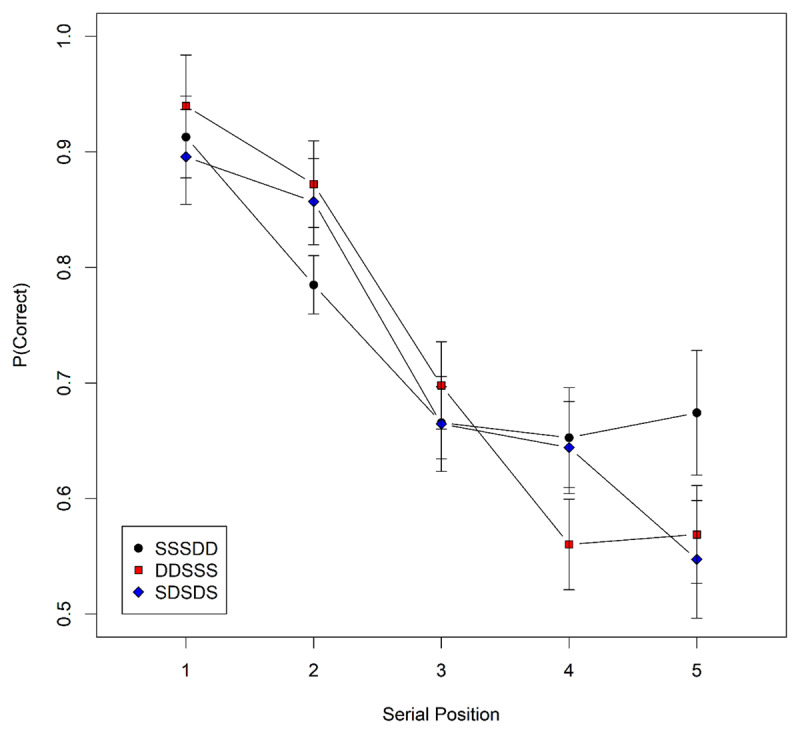
Observed serial-position curves for the three conditions of Experiment 1. Error bars are 95% confidence intervals for within-subject comparisons (Bakeman & McArthur, 1996).

***Figure 2*** shows the proportion of items recalled in their correct position for the three list types. Across all three list types, S words were recalled less well than D words, confirming that participants encoded the words phonologically. Nevertheless, there was no discernible difference in the recall success of the third word between the list types.

We analyzed the proportion of correct responses with a Bayesian general linear model (GLM) with condition, serial position, and similarity (S vs. D) as well as all their interactions as predictors, using the *BayesFactor* package (Morey & Rouder, 2015) for R (R_Core_Team, 2020). The model included random effects of subjects, and random slopes of the three main effects.^2^ We determined the evidence for or against effects of interest by a series of pair-wise model comparisons between a reference model and a constrained version in which we removed the effect of interest. We started with the full model as reference model, first removing the random slopes, then the three-way interaction, followed by each of the two-way interactions, and finally the main effects. After each comparison we maintained the model with more support from the Bayes Factor (BF) as reference model for the next comparison. In this way we obtained BFs for or against individual effects, tested in the context of a model that includes all higher-order effects that are supported by the data. The BF is the ratio of the marginal likelihoods of the two models compared with each other. Throughout, a BF > 1 indicates evidence for the reference model, and BF < 1 indicates evidence for the constrained model. Thereby, BF > 1 reflect evidence for the effect in question, and BF < 1 reflect evidence against it. BF between 1 and 3 are usually regarded as weak evidence, BF between 3 and 20 as positive evidence, BF between 20 and 150 as strong, and BF > 150 as very strong evidence (and analogously for 1/BF when the evidence is against the effect) (Kass & Raftery, 1995).

The random slopes of the three main effects received strong support, BF = 9.6 × 10^45^. The evidence concerning the three-way interaction was ambiguous (BF = 0.7), but as we see no good interpretation for it, and prefer parsimony, we decided to drop it for further comparisons. There was clear evidence against each of the two-way interactions (BF for Similarity × Condition = 0.02; Similarity × Serial Position = 0.07; Condition × Serial Position = 0.008). The main effects of serial position (BF = 5.9 × 10^21^) and for Similarity (BF = 9.0 × 10^6^) were unambiguously supported; the main effect of Condition was not (BF = 0.14). The clear evidence against the two-way interaction of similarity with condition shows that the detrimental effect of similarity was independent of the list composition, that is, independent of whether an item was preceded (or followed) by similar or dissimilar items. A Bayesian ANOVA zooming in on the third list position yielded ambiguous evidence about the effect of condition, BF = 0.7. With a one-tailed test (Morey & Wagenmakers, 2014) of the directed prediction that performance is better in the DDSSS than the SSSDD condition, the evidence remained ambiguous with BF = 1.4. The posterior effect estimate had a mean of 0.02, with 95% credible interval = [–.01, .06], which clearly excludes the effect size of .13 predicted by SOB-CS. A further one-tailed test of the prediction SDSDS > SSSDD yielded moderate evidence against the prediction, BF = 0.24. The 95% credible interval of [-0.03, 0.03] excludes the effect size of .10 predicted by SOB-CS.

The results of Experiment 1 provide no support for the assumption of novelty-gated encoding but also do not present compelling evidence against it, because the Bayes factors for the pairwise comparisons of performance at the critical serial position 3 reflected little evidence either way. Nevertheless, the results are inconsistent with the effect sizes predicted by SOB-CS. One could change the parameters of SOB-CS to make the predicted effect smaller, but that would involve departing from the parameter values that we have consistently used for deriving predictions from SOB-CS in the past.

## Experiment 2

Experiment 2 has been published before with a focus on comparing lists of stimuli from the same category to mixed lists (Farrell & Oberauer, 2013). Here, we report a new analysis of those data to test the prediction from novelty-gated encoding that has not yet been assessed. We presented mixed lists of very dissimilar items for forward serial recall. The mixed lists consisted of two nonwords, two spatial locations in a 5 × 5 grid, and two abstract drawings presented in random order. The experiment also included pure lists of stimuli from one class only, and Farrell and Oberauer (2013) reported that memory for mixed lists was better than memory for pure lists, as predicted from the assumption of interference in SOB-CS and other models. This finding confirms that the similarity manipulation had an effect on memory.

Novelty-gated encoding entails the following prediction for the mixed lists: For any list position *p*, the accuracy of recall in that position should be lower if the other item from the same stimulus class preceded *p* than if it followed *p*. In other words, the first item of a pair from the same class should be recalled better than the second item from that class, holding serial position constant.

### Results and Discussion

**Figure 3:**
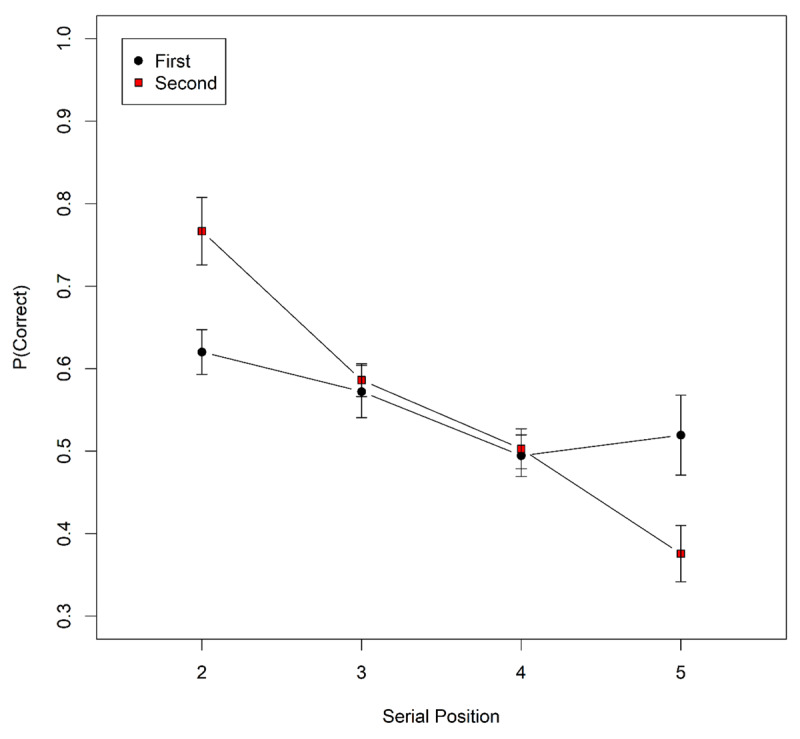
Serial-position curves for Experiment 2. The condition legend indicates whether the item was the first or second item in its category presented on a list. Error bars are 95% confidence intervals for within-subject comparisons (Bakeman & McArthur, 1996).

***Figure 3*** plots accuracy serial position functions according to whether items were the first or second in their stimulus category. Serial position 1 is excluded as it cannot contain the second item from a category pair; equally, serial position 6 is excluded as it cannot contain the first item in a pair. There was clearly no overall benefit for being the first stimulus within a category, contrary to the prediction from novelty-gated encoding.

One explanation for the apparent interaction in ***Figure 3*** is that the item in the “second” condition at serial position 2 was necessarily preceded by an item from the same category. Similarly, the item in the “first” condition at serial position 5 was necessarily followed by an item from the same category. Accordingly, the apparent interaction in the figure could plausibly be driven by an advantage to both items in a pair if they were presented in immediate succession (i.e., an immediate repetition of stimulus class). To account for this possibility, the data were fit with a Bayesian GLM model with serial position, within-category position, and adjacency as factors, the last factor coding whether or not that item was immediately preceded or followed by its same-category partner. A term was also included for the within-category position × serial position interaction. Following Experiment 1, the model included random effects of subjects on intercept, and random slopes for the main effects. Comparing that model with a reduced model excluding within-category position revealed evidence against the main effect of category position, BF=996. Further reducing the model by excluding adjacency revealed evidence for a positive (.086) effect of adjacency on accuracy, BF = 6.24 × 10^7^, so that factor was retained. Further model reduction found some evidence against the interaction, BF = 5.12.

The evidence against the main effect of within-category position in Experiment 2 is evidence against novelty-gated encoding, which should produce poorer performance on the second (and thus less novel) item in the pair. In addition, the positive effect of the adjacency (vs non-adjacency) of members of the category pair also speaks against the contribution of novelty-gated encoding.

A question for future research is the origin of this reinforcing effect of immediate repetition. The effect is analogous to the specifically enhancing effects of direct repetition of items (Henson, 1998). Henson explained that effect as arising from the application of an “immediate repetition” tag. With exact repetitions, such a tag could help because people can get both items correct by remembering just one item plus the tag, but it is less clear how such a tag would help here when both items in the category repetition still need to be remembered.

## Experiment 3

With Experiment 3 we test a prediction from novelty-gated encoding for the complex-span paradigm. Participants tried to remember lists of five consonants, and after presentation of each consonant they read one or several words aloud as the distractor task. In the SOB-CS model, distractors that must be processed are obligatorily encoded into WM by binding them to the position of the preceding item (Oberauer et al., 2012). The strength of distractor encoding is novelty gated. When a processing episode between two consonants consists of several dissimilar words, each word is strongly encoded, whereas when several similar words are to be read, only the first is encoded strongly, and all subsequent words more weakly. Therefore, interference from the distractors should be less severe when the words in a distractor episode are similar to each other than when they are dissimilar. In the extreme case of similarity, when the words to be read are all identical, they should not interfere more than when a single word is to be read after each list item. Lewandowsky et al. (2010) have confirmed this prediction for the extreme case, contrasting distractor episodes of a single word, of four identical words, and of three dissimilar words.^3^ Serial-recall performance was equivalent for the single-word and the four-identical-word condition, but worse when people had to read four different words.

This result, however, is open to two alternative explanations. First, although reading one word four times and reading three different words took the same amount of time to execute, the former could be argued to engage central attention for a shorter time, so that attention would be free to refresh memory items for a longer time (Plancher & Barrouillet, 2013).

Second, the rationale of the design of Lewandowsky et al. (2010) is that each time a word is spoken aloud, it is automatically encoded into WM, so that speaking the same word four times means that it is encoded four times (if only weakly due to novelty gating). More generally, each execution of a distractor operation is considered an event that is obligatorily encoded into WM. This is not a necessary assumption. An alternative view is that what is encoded into WM is not a record of each distractor-processing event, but rather a representation of the information needed to do the distractor task. If that is the case, then reading three different words requires encoding three words into WM, whereas reading the same word four times only requires that word to be encoded once. Hence, the amount of interference depends on how many different words need to be read, regardless of how often each word is repeated.

In light of these considerations, a more diagnostic test of the effect of novelty gating on distractor interference is to compare distractor episodes with several dissimilar words to episodes with the same number of more similar – but still different – words. This is what we did in Experiment 3. The experiment involved four conditions: (1) Reading a single word aloud after presentation of each list item; (2) reading the same word aloud four times; (3) reading four phonologically similar words aloud; and (4) reading four dissimilar words aloud.

### Method

#### Participants

Thirty young adults from the University of Bristol community took part in a one-hour session. The experiment was carried out in agreement with the guidelines of the Faculty of Science Human Research Ethics Committee at the University of Bristol.

#### Materials

For each trial five consonants were sampled without replacement to form the memory list. The distractors were monosyllabic English words. We constructed two sets of 80 words, each containing 20 subsets of 4 rhyming words. Every second participant was assigned one set of words for the practice trials and the other set for the test trials.

The experiment was subdivided into three blocks of 16 trials that contained four trials of each condition in random order. Within each block, the distractors were chosen as follows: Across the four trials of each condition there were 20 distractor episodes to be filled. In a first step, the 20 rhyming subsets were ordered at random for each participant, block, and condition to form a 20 × 4 matrix with one rhyme subset per row. For Conditions (1) and (2), the 20 rhyme groups were assigned to the 20 distractor episodes in the order of the matrix rows, and a single word from each rhyme set was chosen. This choice rotated over groups of four participants: Participant 1 received the first word from each rhyme subset, Participant 2 the second word from each rhyme set, and so on. For Conditions (3) and (4), which required four words per distractor episode, the order of the words in each rhyme group was randomized for each participant, condition, and block. In Condition (3), rhyme groups were assigned to distractor episodes in the order of the matrix rows, so that words were similar within a distractor episode but dissimilar between distractor episodes. In Condition (4), distractor episodes were filled by going down the columns of the matrix in groups of four words, looping through the four columns in order. In this way, the 20 words within and between the distractor episodes of a trial were all dissimilar.

#### Procedure

At the beginning of each trial the condition was announced by displaying “1”, “4 same”, “4 rhyming” or “4 different” in blue for 2 s. This was followed after 0.5 s by a central fixation cross for 1.5 s. The first to-be-remembered letter was presented immediately after offset of the fixation cross in the screen center in red for 0.9 s, followed by a 0.1 s blank screen. Then each distractor word of the following episode was displayed for 0.75 s in the screen center in black, followed by 0.25 s of a blank screen. Participants were instructed to read each word aloud, and their speech was recorded. After the last distractor word in the 5^th^ processing episode, recall was prompted by a question mark, and participants were required to type the letters in their order of presentation. After a 3 s inter-trial interval the next trial commenced.

### Results and Discussion

**Figure 4:**
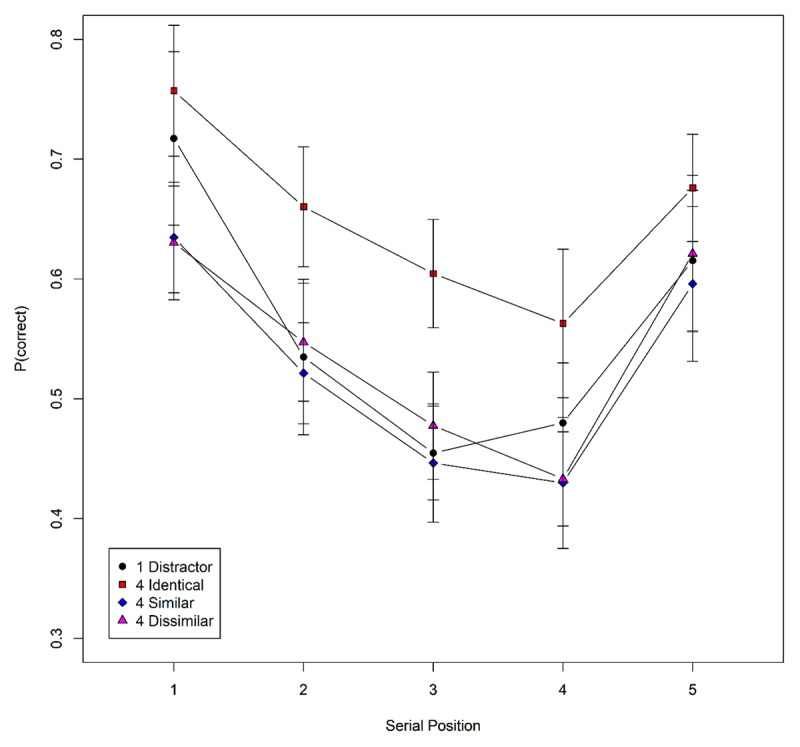
Serial-position curves for the four conditions of Experiment 3; the condition legend indicates the number of distractors following each memory item, and the similarity among distractors. Error bars are 95% confidence intervals for within-subject comparisons (Bakeman & McArthur, 1996).

***Figure 4*** shows the proportion of items recalled in their correct list positions as a function of condition and serial position. We analyzed these data with a Bayesian GLM with serial position and condition as predictors, a random intercept, and random slopes for the two main effects. There was evidence for both main effects (BF_10_ for serial position = 9.8 × 10^12^; for condition = 1.4 × 10^4^) but against their interaction (BF_10_ = 0.02). We then zoomed in on the pairwise comparisons of conditions that are of theoretical interest, applying GLMs with serial position and condition on the respective subset of data.

The critical test for novelty-gated encoding is the comparison of four similar vs. four dissimilar distractors. There was no evidence for a difference between these conditions – the evidence was more in favor of a null effect (BF_10_ = 0.25). As SOB-CS makes a directed prediction, we can calculate the one-sided BF comparing the model with a positive effect of distractor similarity to a null model in which the effect is zero or negative (Morey & Wagenmakers, 2014); this revealed a BF_10_ = 0.12 in favor of the one-sided effect, or 1/0.12 = 8.5 against it.

Memory with a single distractor word was worse than when the same distractor word had to be repeated four times, BF_10_ = 132. This result is surprising as Lewandowsky et al. (2010) have found approximately equivalent performance for these conditions in three experiments. One relevant difference between their experiments and ours pertained to the timing of distractors: In the experiments of Lewandowsky and colleagues all distractors of an episode were presented in rapid succession at the beginning of the episode, and participants were required to read them as quickly as possible. As soon as they finished speaking, the experimenter triggered the presentation of the next list item. In contrast, in our experiment there was a constant 1 s inter-word interval. Therefore, in the current experiment there were potential periods of free time within processing episodes during which participants did not have to do anything, and the total amount of free time was longer when the same word had to be spoken four times than when it was to be spoken once.^4^ Free time between list items can be used to strengthen memory for the items (De Schrijver & Barrouillet, 2017; Oberauer & Lewandowsky, 2019), and this could explain why performance was better with more identical distractors to be spoken. Indeed, a similar beneficial effect of longer episodes of very similar (non-verbal) distractors has been observed before (Oberauer & Lewandowsky, 2014).

Compared to four identical distractors, reading four different distractors – whether similar to each other or not – led to worse serial recall: BF_10_ = 685 for identical vs. similar, and BF_10_ = 154 for identical vs. dissimilar.

## General Discussion

We tested three predictions for serial recall performance derived from novelty-gated encoding, and in all three cases the predictions were not supported by the data. The similarity of a list item to preceding items did not affect its chance of being recalled, neither for similarity within a class of stimuli (Experiment 1) nor for similarity between stimulus classes (Experiment 2). The similarity of distractors in a complex-span task also did not affect the degree to which these distractors interfered with recall of the memory list (Experiment 3). We conclude that it is time to question the assumption of novelty-gated encoding, at least for models of serial recall.

This is a setback for theorists because novelty-gated encoding contributed to explanations of three serial recall phenomena. First, novelty gating naturally generates a primacy gradient of encoding strength that contributes to the explanation of the extended primacy effect in serial-order memory tasks (in contrast to other models that simply assume the presence and form of a primacy gradient). Second, novelty-gated encoding contributed to the prediction from the SOB-CS model that processing series of different distractors impairs memory more than processing the same distractor repeatedly for the same duration (Lewandowsky et al., 2010; Lewandowsky et al., 2008). Third, novelty-gated encoding led to the prediction of the mixed-similarity benefit (Farrell & Lewandowsky, 2003). We should try to find alternative explanations for these phenomena.

An alternative explanation for the primacy effect is that a primacy gradient of memory strength is set up at encoding regardless of the items’ similarity. Such a primacy gradient could arise from cumulative rehearsal, through which list-initial items would receive most rehearsals. Whereas this might occur in certain circumstances, particularly when items are presented at a slow pace, this suggestion is unlikely to provide a comprehensive explanation of how a primacy gradient is generated. The extended primacy effect is undiminished when items are presented too fast for cumulative rehearsal (Tan & Ward, 2008), and it is hardly, if at all, diminished by concurrent articulation (Hughes & Marsh, 2017; Jones, Macken, & Nicholls, 2004; Spurgeon, Ward, & Matthews, 2014). A variant of this explanation could appeal to cumulative refreshing – directing attention to list items in their order of presentation – as causing the primacy effect (Oberauer & Lewandowsky, 2011). That explanation is unlikely, however, because there is no evidence that people refresh items spontaneously at all, let alone refresh them in cumulative fashion (Oberauer, 2019).

Another possible mechanism is that initial list items are encoded most strongly because their representations suffer less inhibition from already encoded items in WM (Grossberg & Pearson, 2008; Manohar, Zokaei, Fallon, Vogels, & Husain, 2019). For instance, in the connectionist model of Manohar and colleagues each item is encoded by binding all its features to a binding unit. The primacy effect arises because binding units compete with each other through lateral inhibition in the binding layer; later list items suffer more competition from the binding units engaged by earlier list items. This idea is similar to novelty-gated encoding, but does not link the amount of inhibition to similarity.

There is some evidence that the primacy gradient is under the person’s control: When people expect an item-recognition test and then need to recall a list in forward order on a small subset of trials, the primacy effect is much diminished compared to when they expect a serial recall test (Duncan & Murdock, 2000). Palladino and Jarrold (2008) compared serial-position effects in regular serial-recall trials with lists of length *n* to those from a running-memory task in which participants had to recall the last *n* items of a list of unpredictable length. On some running-memory trials the presented list ended after *n* items, so that participants had to recall the entire list. Nevertheless, the primacy effect in those running-memory trials was much smaller than in the regular serial-recall trials of the same length. These findings suggest a functional explanation for the primacy gradient: It is set up to facilitate reproduction of the list in forward order (Grossberg & Stone, 1986). Such an explanation is compatible with a mechanistic one, for instance through the build-up of inhibition between representations in working memory, but requires that the mechanism can be modulated by the person’s goal.

In the introduction to Experiment 3 we already foreshadowed an alternative explanation for the effect of distractor variability on complex-span performance: Distractor processing adds to interference not because each processing event is encoded into working memory. Rather, to carry out a distractor task people need to temporarily establish those representations in working memory that they need to control their cognitive operations for that task. A single representation of a word is enough to speak it once or several times in a row, and therefore repeating it several times does not create more interference than saying it once. In contrast, speaking several different words – whether they are similar to each other or not – requires placing each of these words into working memory, so that the amount of interference increases with the number of distractor operations.

An alternative explanation for the mixed-list benefit to recall of dissimilar items is harder to find. Without novelty-gated encoding, current mathematical models of serial recall can produce recall of dissimilar (D) items on mixed lists that is nearly as good as on purely dissimilar lists, but not better (Botvinick & Plaut, 2006; Lewandowsky & Farrell, 2008a; Page & Norris, 1998). We can offer only a speculative possible mechanism for explaining the mixed-list benefit. The similar (S) items on mixed lists studied so far were always rhyming consonants from a small, repeated set. Participants are likely to notice that these items belong to the same phonological category, whereas the D items, which have no common phonological features, are unlikely to be categorized together. If the information encoded about an item includes its category membership, then the rhyme category would be associated to every position of an S item, whereas no category information would be associated to the position of the D items. During recall, positions would be used as retrieval cues for the items. The positions of S items would cue both the identity of the S item in that position as well as the rhyme category. The rhyme category would make the S item more confusable with other S items – thereby contributing to their relatively poor recall in the correct position – but could also be used to block retrieval of D items. In this way, D items on mixed lists would be prevented from being erroneously recalled in positions of S items, and that would increase the chance that the D items are recalled in the correct position. This would not be the case for the same D item in a purely dissimilar list.

To conclude, the idea of novelty-gated encoding has helped to move the field forward: It has led us to correctly predict, and thereby to discover two phenomena: The recall advantage of dissimilar items in mixed-similarity lists, and the smaller detrimental effect of constant than of variable distractor operations in complex-span tasks. Nevertheless, the evidence against novelty-gated encoding is not easily dismissed. It is now time to consider retiring the idea.

Whereas the present investigation has focused on the assumption of novelty-gated encoding in serial-recall tasks, our findings are likely to have implications for memory more broadly. In recent years, the idea that prediction error determines not only the updating of associative strengths in contingency learning, but also the strength of learning more generally, has gained traction (Greve et al., 2017; Rouhani et al., 2018; Trapp, O’Doherty, & Schwabe, 2018). The idea is that episodic memory forms a stronger trace of events that are predicted more poorly. The notion of novelty-gated encoding is a special case of this general idea, applied to memory for lists of randomly ordered stimuli, for which the only basis for prediction of the next stimulus is whatever the preceding stimuli have in common. Lists of similar stimuli have more in common, and therefore provide a stronger basis for predicting the next stimuli, as long as they continue to be similar to the preceding ones. Our experiments can be interpreted as tests of the prediction-error assumption applied to immediate memory for lists. Therefore, the present findings could be understood as a prediction failure of the idea of prediction-error driven memory acquisition, pointing to boundary conditions for that notion.

## Data Accessibility Statements

All data reported in this article are available on the OSF: *osf.io/7cjxv/*

## Footnotes

Relative minimum edit distance (Wagner & Fischer, 1974) with letters as units based on the phonological DISC code provided by Webcelex.

A model also including random slopes of the 2-way interactions led to very unstable BF estimates and was therefore not considered.

Three dissimilar words were compared to four identical ones because overall reading time was equivalent for these conditions, thereby holding the retention interval constant.

For comparison, the mean reading times of distractor words in Lewandowsky et al. (2010), Experiment 3, were 1.15 s for one word, and 2.45 s for 4 identical words. Assuming the same time was needed in our experiment, the expected free time for 1 word would be –0.15 s, whereas for 4 identical words it would be 1.55 s.
